# The Realization of Portable MRI for Indigenous Communities in the USA and Canada

**DOI:** 10.1017/jme.2024.159

**Published:** 2024

**Authors:** Shana Birly, Angela Teeple, Judy Illes

**Affiliations:** 1: TUFTS GRADUATE SCHOOL OF BIOMEDICAL SCIENCE, BOSTON, MA, USA; 2: UNIVERSITY OF MINNESOTA, MINNEAPOLIS, MN, USA; 3: UNIVERSITY OF BRITISH COLUMBIA, VANCOUVER, BC, CANADA; 4: NEUROETHICS CANADA, VANCOUVER, BC, CANADA

**Keywords:** Neuroimaging, Health Equity, Neuroethics, Diversity, Neurodegeneration, Indigenous

## Abstract

The paucity of existing baseline data for understanding neurologic health and the effects of injury on people from Indigenous populations is causally related to the limited representation of communities in neuroimaging research to date. In this paper, we explore ways to change this trend in the context of portable MRI, where portability has opened up imaging to communities that have been neglected or inaccessible in the past. We discuss pathways to engage local leadership, foster the participation of communities for this unprecedented opportunity, and empower field-based researchers to bring the holistic worldview embraced by Indigenous communities to neuroimaging research.

## Introduction

Advances in magnetic resonance imaging (MRI) that are moving scanning capabilities from a fixed environment to one that is mobile and portable (pMRI) has opened up benefits to communities that have been neglected or inaccessible in the past. For these benefits to be realized, however, clinicians and researchers must engage local leadership, achieve buy-in at the community level, and appreciate the values and priorities of populations of interest. For people of Indigenous backgrounds, however heterogeneous they may be, holism and data sovereignty are anchoring principles. Here we explore ways to achieve the embodiment of these principles into imaging studies of the brain where pMRI capabilities, opportunities, and challenges are unfolding.

We focus on Native American People in the USA and Indigenous People in Canada (First Nation, Métis, Inuit) in this paper. We acknowledge and respect the profound heterogeneity of these groups, their different experiences with colonization throughout history, and their treatment in the modern era. We recognize that the specific inclusion of people outside of North America, such as the Såmi of the north, Māori in New Zealand and Aboriginal Australians, are out of scope. For the purposes of this paper, however, we do not differentiate among North American groups, nor do we feel that we are neglecting the world stage, as we view that the most pressing early issues for pMRI to which we speak here are cross-cutting. We focus on issues that directly impact healthcare and research access. We explore how neuroimaging can be tailored and implemented to meet many of the needs and priorities of historically neglected Indigenous communities that may apply within and outside North America and to begin to overcome, or at least address, barriers that have been exacerbated by geographic and socioeconomic factors. In doing so, we strive to contribute to ongoing efforts to rectify inequalities in research and ultimately clinical medicine that have been shaped by a complex history.

We explore how neuroimaging can be tailored and implemented to meet many of the needs and priorities of historically neglected Indigenous communities that may apply within and outside North America and to begin to overcome, or at least address, barriers that have been exacerbated by geographic and socioeconomic factors. In doing so, we strive to contribute to ongoing efforts to rectify inequalities in research and ultimately clinical medicine that have been shaped by a complex history.

Our overall approach to this paper considers, therefore, that before colonization, there were no borders between the Indigenous peoples of North America and current early discourse about pMRI can be a baseline for both groups. There are mentions throughout this paper of data on Native Americans in the USA because there are federally collected data (U.S. Census) that provide insights on the medical landscape of both rurally located Indigenous people and those residing in urban areas. At present, many Indigenous People in Canada are still unrecognized and are fighting for their sovereignty, rights, and access to equal healthcare. Canada’s Truth and Reconciliation Call to Action is the country’s contemporary response to the Indian Act that governed the country’s approach to Indigenous People and Indigeneity since 1876.^1^ As governance of individual Indigenous nations in both the U.S. and Canada will have different sovereign government structures, researchers must understand the legal and regulatory landscape governing the nations with whom they seek to work.

## I. Advances in MRI

The discovery of MRI in the late 1970s led to powerful new opportunities for both research and advances in clinical medicine. For the brain, MRI yields high-resolution images of structures, white matter tracts, lesions, and arteries, enabling the visualization of both healthy neuroanatomy and neurological abnormalities.^2^ Functional MRI, alone and in combination with other imaging methods, yields detailed signals of blood flow and oxygenation associated with a range of applications from resting state, movement, to cognition and executive decision-making.

Historically, MRI has been situated in urban locations for clinical purposes and in academic centers that can afford them for research. For patients and research participants, access has been limited by geography on the one hand, and by costs and wait times on the other. MRI scanners cost millions to purchase, not including siting and the expertise required to interpret scans.^3^ These instruments are expensive to add to Radiology departments, and it is no surprise that MRIs are disproportionately distributed around the world. In high income countries such as the USA and Japan for example, there are 35.3 and 19.5 MRI units per million population. In Canada, the ratio is only 4.6 MRI systems per million. For some regions, especially in rural and remote areas, Radiology departments are often limited to x-ray and other basic clinical imaging capabilities. In the past, MRI for them has been completely out of reach.

Currently, the Indian Health Service (IHS) website reports only 13% of hospitals have an MRI unit in the USA.^4^ According to the Government Accountability Office, the IHS may contract radiological services if they cannot provide them. Barriers to accessibility exist in receiving this contracted healthcare. However, an IHS facility may not be able to afford it without patient insurance, and physicians may be unwilling to provide contracted care at a discount.^5^

There is no similar health entity in Canada for Indigenous People. Federal, provincial, and territorial levels of government share some degree of jurisdiction in the complex patchwork of Canadian policies, legislation, and relationships. Indigenous peoples are included in the per capita allocations of funding from the federal fiscal transfer and are entitled to access insured provincial and territorial health services as residents of a province or territory. Indigenous Services Canada funds or directly provides services for First Nations and Inuit Peoples that supplement those provided by provinces and territories, including primary health care, health promotion, and supplementary health benefits. Indigenous Services Canada directly provides certain health care services to communities and funds the provision of certain community health programs for Inuit people living in Inuit Nunangat, in addition to federal funding provided to territorial governments. Indigenous Services Canada also funds non-insured health care benefits to eligible First Nations and recognized Inuit regardless of where they live in Canada.^6^

## II. The pMRI Revolution

*Building on advances*: Conventional MRI was an advancement in engineering, science, and medicine, and pMRI signifies a further revolution, in particular for people for whom these scans have not been accessible for health care, and without which neuroimaging research with high resolution has been all but impossible. The portability of pMRI eliminates the need for built infrastructure, and low power requirements allow it to be operated with fewer energy concerns. Field strength of different systems, image quality, and the range of available procedures all vary compared to fixed MRI systems.^7^

*Addressing disparities and racism in neurologic health*: The ability to diagnose and triage neurological conditions is paramount to treating and delivering good outcomes for patients, and rurally located Native Americans in the USA and Indigenous People in Canada are particularly prone to diagnostic errors both with, when available, and without access to MRI. Indeed, studies on the Indigenous populations have indicated that individuals from Indigenous backgrounds are less likely to receive specialty care than non-Indigenous people.^8^ Among Native Americans in the USA younger than 65 years, for example, rates of neurological conditions are more than three times higher than that of Caucasians of similar age.^9^ In addition, individuals who are American Indian/Alaska Native (AIAN) have poorer overall survival rates and comorbidities from brain and other central nervous system tumors as compared to individuals who are Caucasian.^10^ Moreover, among the large number of individuals identifying as American Indian or Alaska Native in the USA, almost 15% of these individuals remain uninsured. This number is almost double the U.S. national average (8.4%), and 2.4 times larger than the Caucasian uninsured percentage (6.2%).^11^ In Canada, 5% of the population is Indigenous. Although the healthcare system in Canada is nationalized, Indigenous peoples face a lack of health services, particularly in remote communities. Indigenous peoples in both countries experience anti-Indigenous racism in health systems, and a lack of cultural safety and acceptance of Indigenous health and healing models. Health insurance access, location of reservations, and tribal government finances are all factors for Indigenous People in North America that impact access to quality healthcare. Life expectancies for AIAN in the U.S. are 5.5 years less than Caucasians in the U.S. (73.0 years to 78.5 years, respectively).^12^ Reduced life expectancy for Indigenous populations emphasizes why access to all healthcare capabilities, including MRI, is not only an imperative but a human right.

In order to combat the health disparities within Native American communities, the Indian Health Service (IHS) was created to be accessible to all enrolled members of federally recognized tribal nations in the USA. The Indian Health Care Improvement Act of 1976 (25 U.S.C § 1601)^13^ and the Snyder Act of 1921 (25 U.S.C § 13)^14^ comprise the basic legislative authority for the IHS and how healthcare is managed on reservations, trust land, and lands owned by tribal governments. Although the IHS was created to combat healthcare barriers for Native Americans, access to these services is affected by the wait time between making an appointment and the delivery of a service, travel distance to facilities, and lack of transportation. If a healthcare location is close enough for a tribe to use, barriers continue to exist in the quality of care and the capabilities of that facility since not all clinics have the equipment and level of health specialists necessary.

*Recognizing sovereignty over care and research:* Sovereignty over health care on the lands of Indigenous People is essential in medicine; data sovereignty is essential in research. The approach to portable MRI, therefore, may be as similar as it is unique because both aspects of sovereignty must be put to work in tandem.

When establishing care models for Indigenous peoples, consultation and discussion are imperatives to ensure that beliefs and approaches to traditional medicine and healing are accounted for, as is an understanding of patient autonomy by healthcare personnel.^15^ Amongst the hundreds of nations across the United States and Canada, the vast diversity in cultures, traditions, and beliefs has, alongside it, traditionally diverse approaches to medicine and healing that are part of all Indigenous health systems. Two-eyed Seeing has been advanced as one way in clinical neuroscience to achieve this goal.^16^ As appropriate, they must be integrated in approaches to healthcare on traditional lands.

Beyond an appreciation of traditional, biomedical, and hybrid perspectives on health and care, the benefits of early detection of disease for better health outcomes, especially among minoritized populations, cannot be ignored. While it is beyond the scope of this paper to provide a detailed comparative analysis, we note that there is a significant, yet under-documented divide in healthcare outcomes not only between rural and urban areas but also across different racial and ethnic groups within rural America due to lack of trust and access to disease prevention and detection strategies.^17^ To our knowledge, the data specifically comparing health outcomes between Indigenous populations and white rural patients are markedly limited.

There is no overstating the importance of respect for the differences in priorities for research, data collection methods, and dissemination of results of studies that involve Indigenous communities. At the outset, to build trust within the community among researchers and participants, partnerships must be formed at the regional level with elders, elected tribal officials, and community members as consultants who are keen to provide perspective. There should no longer be any tolerance for helicopter methods in which community members are merely passive subjects.^18^ Management for both expected and unexpected findings require active engagement and planning.^19^ Creative communication strategies that can reliably reach participants irrespective of their technological resources must be established, especially as many patients in rural areas live outside of reliable cell phone or internet coverage and often lack regular access to phones or computers.

Many Indigenous-led initiatives and governance structures provide guidance for research and are setting the example for data sovereignty today. The Native BioData Consortium in Eagle Butte, SD, for example, was created by Indigenous scientific and bioethics experts to keep Indigenous research samples and data within the governance of Indigenous communities. In Canada, OCAP® (Ownership, Control, Access, and Possession) was established to protect the rights of Indigenous People to ensure ownership and control of their information, decision-making that is powered by their lifeways, priorities, and concerns.^20^

With growing research spaces on tribal lands like the Native BioData Consortium, it is pertinent to consider the ownership of data as being as sovereign as the subjects that created it.^21^ Researchers can navigate issues of consent and confidentiality in Indigenous contexts by recognizing and respecting that many Indigenous groups have a collective ethic, and decisions may be made by group consensus or through a council of defined authority. Researchers must engage with the various forms of representation chosen by the community and think differently about data governance beyond individual consent. As such, researchers should consider alternative models of data management, such as Indigenous-led bio-data repositories, that prioritize Indigenous sovereignty in research and data.

Researchers must adhere to ethical guidelines and regulations at a federal level for the protection of human subjects, as outlined in the Code of Federal Regulations, while also considering the specific cultural and ethical considerations of the tribal community.^22^ This guidance, while codified within the USA, also sets a precedent that should inform research practices globally, particularly when involving Indigenous communities. In doing so, researchers must integrate not only these federal regulations but also consider the specific cultural and ethical considerations of each Indigenous community. At the local level, researchers should be aware whether or not the nation has a tribal Institution Review Board (IRB) process and the extent to which it is formally instituted similar to non-tribal IRBs, or more community-based where tribal members are invited to discuss the research. Researchers should always familiarize themselves with the requirements of any process and the cultural importance of these ways going forward.

International legal agreements also provide a context for these ethical considerations. The 1948 Universal Declaration of Human Rights supports the equal opportunity for all individuals to access the fruits of scientific advancement in Article 27.1.^23^ The 1966 International Covenant on Civil and Political Rights, through Article 1.1, explicitly affirms the right of all peoples to self-determination.^24^ The 1966 International Covenant on Economic, Social, and Cultural Rights, in Article 12.2.d, emphasizes the right to equal access to medical services and medical attention.^25^ Collectively, these international agreements underscore the importance of respecting and integrating Indigenous rights into research practices, ensuring that Indigenous communities can partake fully and fairly in scientific advancements.

Building on these foundations, the United Nations Declaration on the Rights of Indigenous Peoples (UNDRIP) specifically addresses these principles through its provisions. Article 31 of UNDRIP advocates for the rights of Indigenous peoples to maintain, control, protect, and develop their intellectual property over their cultural heritage and traditional knowledge, which includes data from research involving their communities.^26^ This underscores the critical need for Indigenous-led data governance models that respect and promote Indigenous data sovereignty. Additionally, Article 24 reinforces the right to traditional medicines and health practices, advocating for the inclusion of culturally appropriate medical technologies such as pMRI systems in Indigenous healthcare practices.^27^ Article 3 of UNDRIP emphasizes the right to self-determination, advocating that Indigenous peoples must have a decisive voice in how health technologies and research practices that affect them are implemented.^28^

While international laws may not be directly enforceable, their influence is significantly enhanced when respected and implemented by influential countries such as the USA and Canada. Compliance not only sets a powerful example but also promotes a broader, global respect for the rights of Indigenous populations, encouraging the integration of these principles into national policies and practices.

In complement to the profound work being done within Indigenous communities on data sovereignty, it is beneficial to consider the broader context of international data rights. For instance, the General Data Protection Regulation offers a robust framework for data protection that could inspire similar protections for Indigenous data. The FAIR Data Principles, emphasizing Findability, Accessibility, Interoperability, and Reusability of data, could be adapted to respect and uphold the unique needs of Indigenous data sovereignty, ensuring that data about Indigenous peoples is managed in a way that is both ethical and respectful of their cultural values.

Collectively, international agreements and frameworks underscore the importance of respecting and integrating Indigenous rights into research practices and ensuring that Indigenous communities can partake fully and fairly in scientific advancements.

## III. Capacity-Building and Training

Recruiting primary and allied healthcare professionals to rural and under-resourced communities is a ubiquitous and well-known challenge that critically impacts the structure and efficacy of healthcare systems serving these communities. Advancements in career opportunities in medical imaging, however, may naturally map onto the landscape of the doors opened by pMRI. Indeed, the training of local operators is ripe for opportunity.

Specialist education is needed to become a radiologic technologist (RT) and requires advanced certification or degrees. Today, more than 50% of practicing RTs are Caucasian.^29^ RTs from minority populations are subject to the same certification process as others through the American Registry of Radiologic Technologists, ^30^ without consideration of the economic challenges they may face as they look to gain this professional training. Acknowledging this is essential for the practical deployment and scalability of pMRI technology.

We propose that this gap can be addressed through new opportunities for RTs to gain an introduction to pMRI and certificate or degree programs at tribal colleges and universities (TCUs) that are located near nations. There are 35 accredited TCUs in the USA as of 2022 and in the 2015-2016 academic year, they enrolled approximately 11.2% of all AIAN undergraduate students.^31^ These were created to facilitate nation-building through self-determination,^32^ increase the completion rates of AIAN students, and to counteract the forced assimilation policies and practices of the USA government and religious institutions.^33^ TCUs offer applied science degree programs, and 23 TCUs also provide certificate programs in the health sciences.

In Canada, Indigenous People have tribal colleges and universities known as Indigenous Institutes of Higher Learning (IIHL). There are 26 Indigenous post-secondary institutions in Canada that are dedicated to preserving and teaching Indigenous cultures, languages, and traditions, while also providing academic programs in a variety of fields. These IIHLs vary in size and scope, offering a range of programs and courses, like health sciences, which integrate Indigenous knowledge and perspectives.^34^ They play a crucial role in providing post-secondary education that is culturally relevant and accessible to Indigenous students. Many of these colleges and universities collaborate with mainstream universities to provide accredited programs and these partnerships allow students to receive a recognized degree while studying within an environment that respects and incorporates Indigenous knowledge and perspectives.

TCUs and IIHLs can provide didactic, on-site, and clinical training if a portable MRI can be obtained. By leveraging the proximity of TCUs and IIHLs to tribal areas and nearby health facilities, MRI technologists can be trained close to where the technology will be utilized.

## IV. Giving Back in Research: Duty of Care

There are significant challenges conducting research with populations that have limited access to medical care, and among them is the duty to provide healthcare when that need is detected through research. Richardson and others have argued that giving back this way in research is a moral imperative.^35^ Others uphold the clear philosophical importance of this duty to care but have suggested that such a requirement may have the negative effect of impeding research given uncertainties about who should or can shoulder the ensuing clinical burden, costs, and other practical realities of achieving this goal.

## V. Remuneration

Ethical remuneration consists of both reimbursement for costs incurred by participants (e.g., transportation) as well as compensation for participant time and effort. The value of a financial incentive should not be so great that it distorts a participant’s ability to evaluate the risk of involvement. The exact value should be context-specific and ideally co-determined by researchers together with participants or groups. A surrogate can play a key role in stewarding reimbursement on behalf of a participant with limited capacity and to use it directly in care for example or hold it in trust. For work with Indigenous communities, researchers must consult with Elders to identify both monetary incentives in addition to traditional gifts. When appropriate, cash remuneration is preferred as gift cards and similar tokens can be paternalistic and stigmatizing.

## VI. Sharing of Results and Reciprocity

Beyond remuneration, reciprocity in research prioritizes the participant-researcher relationship, mutual respect, and autonomy.^36^ To achieve reciprocity, participant and community benefit must be considered outside of the envelope of traditional academic products. Models to achieve this goal are through written and digital products (e.g., resource books, infographics, films^37^) in addition to community events for knowledge translation and exchange that give back to the communities that gave of themselves.

## Conclusion

Through the democratization of MRI imaging capabilities to rural areas, Native American reservations, and Indigenous Lands, the ability to certify and energize a tribe’s or nation’s ability to lead in their own healthcare will improve outcomes and broadly meaningful research results. Here, we grounded our discussion in the rights of Indigenous populations and emphasize that practices not only adhere to global legal standards but also deeply respect and promote the intrinsic rights of Indigenous communities.

The expansion of MRI capabilities through its portability signifies a pivotal moment: it is an evolution that not only offers an opportunity to enhance healthcare delivery but ensures alignment with the needs and sovereignty of Indigenous communities. By expanding the training and backgrounds of research and care teams who are resourced to these communities, responsive research policies and public health measures can be developed and adopted. We conclude with five key takeaway points shown in **Table 1**.

**Table 1:** Key takeaways in considering pMRI for neurologic care and research in the neurological sciences.

Democratization of technology	pMRI enables tailored and equitable healthcare and research that respect and uphold the sovereignty and specific needs of Indigenous communities. Continued progress will bridge gaps in access, promote benefits, mitigate risks, and empower Indigenous communities to manage and utilize capabilities in ways that align with their cultural values and health priorities.
**Clinical practice and outcomes**	The roll-out of pMRI for clinical care of Indigenous Peoples must take into consideration traditional medicine, multiple ways of seeing wellness and disease, historical mistrust, and health disparities. It must integrate early detection to close health disparities. Pathways for managing expected and unexpected findings must be put in place prior to the implementation of any scanning protocol. Innovative communication strategies to reach patients in remote areas without reliable internet or cellular service will ensure that all individuals receive timely and effective care.
**Land and data sovereignty in research**	Data sovereignty is at the core of all neuroimaging-related research. Indigenous-led governance models offer pathways for study conceptualization, data management, dissemination of results and giving back to the community that respect and value the heterogeneity of Indigenous Peoples.
**Duty to care**	Any research that may yield clinical considerations for participants should ideally integrate a management plan for follow up.
**Equity and capacity-building**	pMRI represents a new opportunity to build capacity in MRI operations among Indigenous People interested in health research and care. A locally trained workforce will foster sustainability and self-reliance in research and health care practices.

By anchoring our approach in universally recognized rights, and by aligning pMRI practices with these principles, we commit to a framework that is ethical, respectful, and fundamentally supportive of equity and justice for Indigenous peoples. The integration of pMRI technology into Indigenous health care systems for the brain, and in the future for the body, stands as a testament to the power of combining science with social justice, and highlights the transformative potential of culturally informed discovery science and health care.

## Note

Preparation of this article was supported by an NIH/NIMH-funded BRAIN Initiative project titled Highly Portable and Cloud-Enabled Neuroimaging Research: Confronting Ethics Challenges in Field Research with New Populations, Award # 1RF1MH123698. J.I. is supported by the Canadian Institutes of Health Research, Fonds de Recherche du Québec, the Government of Canada New Frontiers in Research Fund (NFRFT-2020-00238), and the North Family Foundation. Any opinions, findings, and conclusions or recommendations expressed in this material are those of the authors and do not necessarily reflect the views of NIH. The authors have no relevant disclosures.
